# Tobacco Treatment Outcomes in Patients With and Without a History of Depression, Czech Republic, 2005–2010

**DOI:** 10.5888/pcd10.130051

**Published:** 2013-09-19

**Authors:** Lenka Stepankova, Eva Kralikova, Kamila Zvolska, Alexandra Kmetova, Milan Blaha, Zbynek Bortlicek, Michal Sticha, Martin Anders, Darrell R. Schroeder, Ivana T. Croghan

**Affiliations:** Author Affiliations: Lenka Stepankova, Eva Kralikova, Kamila Zvolska, Alexandra Kmetova, The 3rd Medical Department, and Institute of Hygiene and Epidemiology, First Faculty of Medicine and General University Hospital, Charles University in Prague, Czech Republic; Martin Anders, Department of Psychiatry, First Faculty of Medicine and General University Hospital, Charles University in Prague, Czech Republic; Milan Blaha, Zbynek Bortlicek, Michal Sticha, Masaryk University, Brno, Czech Republic; Darrell R. Schroeder, Mayo Clinic, Rochester, Minnesota.

## Abstract

**Introduction:**

Higher prevalence of smoking among depressed patients, as well as the risk of depression in smokers, is well documented. The proportion of patients with a history of depression among those seeking intensive treatment of tobacco dependence is also high. In contrast, evidence of treatment success in this subgroup of patients is controversial. The aim of this study was to compare smoking abstinence rates after tobacco treatment in smoker**s** with and without a history of depression.

**Methods:**

We reviewed retrospective data from 1,730 smokers seeking treatment in Prague, Czech Republic. History of depression was defined as past diagnosis of depression or current treatment of depression. After a 1-year, self-reported smoking status was validated by expired-air carbon monoxide. We used logistic regression to analyze associations between abstinence rates, history of depression, and other factors (eg, age, sex, tobacco dependence).

**Results:**

Of 1,730 smokers treated, 289 (16.7%) had a history of depression. The smoking abstinence rate at 1 year was 32.5% for smokers with a history of depression and 38.7% for those with no history (*P* = .048). Among women, abstinence did not differ between groups (35.0% vs 35.7%; *P* = .86). However, among men, those with a history of depression had lower rates of abstinence (27.4% vs 41.3%; *P* = .009). After adjustment for baseline covariates, history of depression was not significantly associated with smoking abstinence in men or women.

**Conclusion:**

Intensive outpatient tobacco treatment programs can achieve abstinence rates among smokers with a history of depression similar to rates among the general population.

## Introduction

Smoking represents a principal cause of preventable premature mortality and morbidity. Thus, over the past few decades interest has grown in various methods of tobacco-dependence treatment and in making sure treatment is more accessible in many countries (1,2), including the Czech Republic, where this study was conducted. The Czech Republic has 41 specialized centers for tobacco dependence (3,4).

The prevalence of smoking is high among patients with psychiatric comorbidity (5). Evidence is growing that smokers who have a mental illness are as ready to quit as other smokers and can quit without any threat to their mental health recovery (6). A 2-way relationship between depression and smoking exists. Smokers are more likely than nonsmokers to be depressed (7), and conversely, depressed patients are more likely than nondepressed patients to be smokers (8). One study showed that 23.7% of current smokers, 14.6% of ex-smokers, and 6.2% of nonsmokers had a history of depression (9). Analysis of 2,139 outpatient smokers in treatment indicated that 54% of female smokers and 31% of male smokers had been previously treated for symptoms of depression (10). Data are not available on the percentage of patients with a psychiatric comorbidity who seek tobacco-dependence treatment in the Czech Republic. The prevalence of depression in European Union countries is 6.9% (11). Treatment success rates among patients with a psychiatric comorbidity vary: if serious, debilitating psychiatric diseases are present (schizophrenia, bipolar affective disorder, and other forms of psychosis), the success rate of tobacco-dependence treatment is lower than in the general population of smokers (12). Results are not unequivocal for patients who have a history of depression. Some reports find no differences in treatment success rates between depressed and nondepressed patients (13); some find slightly to significantly decreased success rates among patients who have a history of depression (14,15). Also controversial are data on relapse to depression in patients who are quitting smoking and have a history of at least 1 depressive episode. In the most recent study of a group of patients with a history of depressive disorder who quit smoking, the number of depressive symptoms in the patients who were able to quit smoking was the same (16) or significantly lower (17) than in those who failed to quit. This recent study contradicts earlier studies (18,19), which described a higher risk of depression associated with smoking cessation. In a meta-analysis, the influence of smoking cessation on depression was unclear (20).

Among smokers seeking treatment, women have had a higher prevalence of past depression than men and also a greater degree of nicotine withdrawal, including depressive symptoms, during abstinence attempts (10). These findings were corroborated by an independent review that focused on smoking cessation treatment in smokers with depression; the review concluded that depression has a greater effect on reducing success rates in women than in men (21). Recommended therapeutic interventions for patients with a history of depression include closer monitoring of potential development of depressive symptoms, consideration of possible drug interactions, and more follow-up visits. In certain countries (eg, United States, United Kingdom, Czech Republic), recommendations and guidelines exist for the treatment of tobacco dependence in patients with comorbid psychiatric diseases (1,22,23). The objective of our study was to compare the success rate of tobacco-dependence treatment, according to sex and history of depression, among smokers seeking treatment at the Center for Tobacco-Dependent in the Czech Republic.

## Methods

### Treatment program

Among 2,044 smokers who sought treatment from 2005 through 2010 at the Center for Tobacco-Dependent at General University Hospital, in Prague, Czech Republic, 1,730 (85%) entered the treatment program and were followed for 1 full year. This study focused on the complete sample of 1,730 smokers. All patients included in the treatment program underwent an intensive face-to-face counseling intervention, which focused on tobacco-dependent smoking cessation. Data were collected as part of the routine operation of our center. Patients could be referred by their physicians or self-referred to our center, which is part of a large university hospital. All patients signed informed consent for treatment and data collection. This study was exempt from ethics committee oversight because it was a retrospective study that used standard-of-care procedures.

For the treatment program, the center used evidence-based procedures included in Czech Republic Clinical Guideline Recommendations, which follows international treatment guidelines (1,24). Treatment was tailored to the needs of the individual, and treatment may have differed for each patient by type of medication used or by the number of patient visits; these variables depended on patient medical history, preferences, and compliance. The course of the initial treatment program and the information collected at the start of treatment followed a similar scheme for all patients even if treatment was managed by various therapists. The center staff who attended all patient visits included a nurse (responsible for measures of expired-air carbon monoxide [CO] and blood pressure and collection of questionnaire data) and a physician (responsible for the treatment program, including psychosocial and pharmacotherapy). All physicians were certified tobacco-treatment specialists and had completed a course developed by the Czech Medical Chamber or the Tobacco Treatment Specialist Certification Program at Mayo Clinic in Rochester, Minnesota.

During the first visit, which took place before entering into the treatment program and which lasted about 1 hour, the degree of tobacco dependency was evaluated, a medical history collected, and a basic physical examination performed. In addition, patients completed an intake questionnaire, which contained information about the patient’s history of tobacco use, including age at first use and previous quit attempts. Information on demographics (ie, education, age, sex, marital status) and past diagnosis and current treatment of depression was also collected. Smokers with current treatment of depression or a past diagnosis of depression were outpatients whose disease was stabilized and who came to our specialized center of their own free will and were actively interested in treatment. We categorized patients as having a history of depression or not having a history of depression. We defined “history of depression” as past diagnosis of depression (diagnoses codes F32.x–F33.x in the *International Classification of Diseases, Tenth Revision* [ICD-10]) or current treatment of depression. Tobacco dependence was defined by using the Fagerström Test for Cigarette Dependence (FTCD) (25).

During the second visit, which we defined as the baseline for the treatment program and which lasted about 2 hours, the therapist and patient discussed physical and psychosocial tobacco/nicotine dependence and coping strategies, including how to manage or avoid situations that might encourage smoking. At this visit, pharmacotherapy was also introduced. At the end of the second visit, the patient and therapist decided the subsequent course of treatment, including pharmacotherapy type and dosage. They also planned a target quit day (the date the patient planned to quit) and the date of the follow-up visit. Czech health insurance covers the intial intervention and 8 follow-up visits per year but not pharmacotherapy, so smokers could avail themselves of the program regardless of their ability to pay. Each follow-up visit included a counseling component that addressed withdrawal, coping strategies, and psychosocial support.

Follow-up visits on average lasted 30 minutes. The first of these was planned for approximately 1 week after the target quit day, and subsequent visits took place in approximately 2-week intervals until the third month of treatment. After the third month, 3 visits took place at an interval of 4 weeks until 6 months from the initial visit. Patients were permitted to codetermine (with their physician) the frequency of their visits. The final visit took place 1 year after the actual quit day (the date when the patient actually quit smoking). Patients who failed to attend the final visit were contacted by telephone. At all visits, vital signs and expired-air CO were measured, changes in the medical history recorded, and data on pharmacotherapy collected.

### Data analysis

A special web-based application was developed to collect and analyze data from the Center for Tobacco-Dependent. Treatment success at 1-year follow-up was evaluated by using the following criteria: a patient was classified as successful if he or she attended the final follow-up visit 1 year from actual quit day and reported continuous abstinence from tobacco products, which was biochemically confirmed through expired-air CO (≤6 ppm) at each follow-up visit. Expired-air CO was measured by a Micro^+^ Smokerlyzer monitor (Bedfont Scientific Ltd, Kent, United Kingdom). No face-to-face visit was required for patients who self-reported through telephone contact that they were using tobacco. Using the intent-to-treat approach, we classified as smokers patients who missed or refused their final face-to-face visit. The evaluation of the outcome was based on Russell Standard (RS) criteria (26). We used the RS criteria because our study was a trial of cessation aids; participants had a defined target quit date, and they had face-to-face contact with researchers or clinic staff according to the following description (26):

1) follow-up for 6 months (RS6) or 12 months (RS12) from the target quit date or the end of a predefined “grace period”; 2) self-report of smoking abstinence over the whole follow-up period allowing up to five cigarettes in total; 3) biochemical verification of abstinence at least at the 6-month or 12-month follow-up point; 4) use of an “intention-to-treat” approach in which data from all randomized smokers are included in the analysis unless smokers died or moved to an untraceable address (participants who are included in the analysis are counted as smokers if their smoking status at the final follow-up could not be determined); 5) following-up “protocol violators” and using their true smoking status in the analysis; and 6) collecting follow-up data blind to smokers’ allocation to trial group.

To assess whether the association between a history of depression and smoking abstinence differed between men and women, a logistic regression analysis was performed and the sex-by-depression interaction evaluated. Because a significant interaction was detected, all subsequent analyses were performed separately for men and women. Baseline characteristics were summarized by using descriptive statistics and compared between those with and without a history of depression by using the rank sum test for continuous variables and the χ^2^ test (or the Fisher exact test) for nominal variables. Multivariable logistic regression analyses were performed to assess whether a history of depression was significantly associated with abstinence at 1 year after adjusting for other baseline covariates (eg, age, sex, FTCD). Findings from the logistic regression analyses were summarized using odds ratios (ORs) and corresponding 95% confidence intervals. In all cases, 2-tailed *P* values of .05 or less were considered significant.

## Results

Of 1,730 smokers treated, 289 (16.7%) had a history of depression. Compared with women who did not have a history of depression, women who had a history of depression at baseline were significantly older, started smoking at a later age, were more dependent on nicotine, and were more likely to be treated with combination therapy (Table 1). Similarly, men who had a history of depression, compared with men who did not have a history, were heavier smokers, more dependent on nicotine, and more likely to be treated with combination therapy. Most patients in our sample (76.9%, 1330/1730) received pharmacotherapy designed for treating tobacco dependence**.** The number of visits did not differ significantly between subgroups.

**Table 1:** Characteristics of Study Participants by Sex and History of Depression, Study on Tobacco Treatment Outcomes, Czech Republic, 2005–2010

Characteristic^a^	Women, No. (%)			Men, No. (%)		
	No History of Depression (n = 669)	History of Depression (n = 194)	*P* Value^b^	No History of Depression (n = 772)	History of Depression (n = 95)	*P* Value^b^
**Age at baseline visit, y**						
≤39	249 (37.2)	50 (25.8)	.006	289 (37.5)	31 (32.6)	.41
40–49	106 (15.8)	32 (16.5)		163 (21.1)	24 (25.3)	
50–59	142 (21.2)	61 (31.4)		157 (20.4)	24 (25.3)	
≥60	172 (25.7)	51 (26.3)		162 (21.0)	16 (16.8)	
**Age at smoking initiation, y**						
≤18	383 (57.3)	91 (46.9)	.01	498 (64.7)	55 (57.9)	.21
≥19	285 (42.7)	103 (53.1)		272 (35.3)	40 (42.1)	
**Fagerström test for cigarette dependence score (FTCD)**						
0–5	267 (50.5)	63 (40.6)	.04	283 (44.4)	26 (31.7)	.03
≥6	262 (49.5)	92 (59.4)		355 (55.6)	56 (68.3)	
**No. of cigarettes per day**						
1–10	56 (8.4)	13 (6.7)	.40	44 (5.7)	3 (3.2)	.02
11–20	362 (54.3)	95 (49.2)		335 (43.7)	28 (29.5)	
21–30	157 (23.5)	54 (28.0)		219 (28.6)	35 (36.8)	
≥31	92 (13.8)	31 (16.1)		168 (21.9)	29 (30.5)	
**No. of prior attempts to quit**						
0	44 (7.2)	12 (6.6)	.94	41 (5.7)	7 (7.7)	.29
1	158 (25.8)	47 (25.8)		209 (28.8)	18 (19.8)	
2–5	358 (58.5)	105 (57.7)		386 (53.2)	53 (58.2)	
≥6	52 (8.5)	18 (9.9)		89 (12.3)	13 (14.3)	
**No. of visits**						
3–5	334 (50.0)	91 (46.9)	.46	407 (52.7)	55 (57.9)	.38
≥6	334 (50.0)	103 (53.1)		365 (47.3)	40 (42.1)	
**Medication**						
No medication	145 (21.7)	42 (21.6)	.02	189 (24.5)	24 (25.3)	.002
Only varenicline	194 (29.0)	52 (26.8)		283 (36.7)	22 (23.2)	
Only nicotine replacement therapy	249 (37.2)	60 (30.9)		224 (29.0)	29 (30.5)	
Other therapy or combination	81 (12.1)	40 (20.6)		74 (9.9)	20 (21.1)	

a Data were available for more than 98% of patients for all characteristics except the FTCD score and the number of prior attempts to quit. For the FTCD score, not everyone completed all the questions needed to calculate the score; 79% completed all questions. For the number of prior attempts to quit, 92% of study participants answered the question

b Continuous characteristics were compared using the Mann–Whitney test. Discrete characteristics were compared using the Fisher exact test or χ^2^ test.

The overall smoking abstinence rate after 1 year was 37.7%. The smoking abstinence rate at 1 year was 32.5% for smokers with a history of depression and 38.7% for those with no history (*P* = .048). Logistic regression analysis indicated that the association between depression and smoking abstinence was dependent on sex (depression-by-sex interaction, *P* = .04). Among women, the smoking abstinence rate did not differ between smokers with a history of depression (35.0%) and smokers with no history (35.7%) (*P* = .86). However, among men, those with a history of depression had lower smoking abstinence rates than those with no history (27.4% vs 41.3%, *P* = .009). After adjusting for baseline covariates, we found no significant associations between a history of depression and smoking abstinence in women or men (Table 2).

**Table 2:** Multivariable Logistic Regression Evaluating Characteristics Associated With Successful Abstinence Among Men and Women After 1 Year, Study on Tobacco Treatment Outcomes, Czech Republic, 2005–2010

Characteristic	Women			Men		
	No. of Patients^a^	OR (95% CI)^b^	*P* Value^b^	No. of Patients^c^	OR (95% CI)^b^	*P* Value^b^
**History of depression**						
No	477	Reference	—	584	Reference	—
Yes	146	0.87 (0.55–1.39)	.57	78	0.57 (0.30–1.10)	.09
**Age at baseline visit, y**						
≤39	213	Reference	—	255	Reference	—
40–49	103	0.93 (0.51–1.68)	.81	140	1.17 (0.69–1.99)	.56
50–59	151	1.67 (0.99–2.81)	.05	136	1.27 (0.75–2.15)	.36
≥60	156	0.88 (0.52–1.50)	.64	131	1.68 (0.98–2.89)	.06
**Age at smoking initiation, y**						
≤18	348	Reference	—	431	Reference	—
≥19	275	1.30 (0.84–2.01)	.24	231	1.26 (0.82–1.94)	.29
**Fagerström Test for Cigarette Dependence score**						
0–5	299	0.89 (0.56–1.40)	.61	284	0.98 (0.63–1.53)	.93
≥6	324	Reference	—	378	Reference	—
**No. of cigarettes per day**						
1–10	45	2.93 (1.11–7.71)	.03	38	2.80 (1.06–7.38)	.04
11–20	339	1.37 (0.72–2.60)	.33	267	2.03 (1.15–3.57)	.01
21–30	149	0.86 (0.45–1.67)	.66	203	1.27 (0.74–2.18)	.39
≥31	90	Reference	—	154	Reference	—
**No. of prior attempts to quit**						
0	38	1.01 (0.35–2.89)	.99	38	1.08 (0.42–2.80)	.87
1	161	1.12 (0.53–2.38)	.77	184	1.40 (0.73–2.67)	.31
2–5	372	0.89 (0.44–1.79)	.73	355	1.04 (0.58–1.88)	.89
≥6	52	Reference	—	85	Reference	—
**No. of visits**						
3–5	314	Reference	—	366	Reference	—
≥6	309	4.58 (2.97–7.05)	<.001	296	7.19 (4.72–10.94)	<.001
**Pharmacotherapy**						
No medication	142	Reference	—	172	Reference	—
Only varenicline	165	5.31 (2.55–11.06)	<.001	224	3.82 (2.10–6.97)	<.001
Only nicotine replacement therapy	228	3.41 (1.69–6.88)	.001	191	1.81 (0.98–3.32)	.06
Other therapy or combination	88	3.97 (1.77–8.87)	.001	75	1.35 (0.63–2.89)	.44

Abbreviation: CI, confidence interval.

a Analysis restricted to 623 women who had information available for all characteristics.

b Odds ratio for successful abstinence; *P* value determined by Wald test. Odds ratio greater than 1.0 indicates increased likelihood of abstinence.

c Analysis restricted to 662 men who had information available for all characteristics.

## Discussion

This study was the first to report on smoking abstinence outcomes by history of depression in smokers from the Czech Republic. Similar to reports from other countries, the overall smoking abstinence rates in the Czech Republic were better in patients who did not have a history of depression than in patients that did. Upon further analysis, however, the difference in rates according to history of depression was significant only in men, contrary to reports from other countries. This finding suggests that depression has a greater effect on the reduction of abstinence rates among women than among men (21).

The relatively high smoking abstinence rate (32.5%) among those who had a history of depression may be explained by our study’s inclusion of stable outpatients. Most (76.9%) patients in our sample received pharmacotherapy designed for treating tobacco dependence; the high success rate may reflect the effect of this therapy. Also, because pharmacotherapy is not covered by health insurance, the patients in our study may have been more motivated to quit than patients unwilling or incapable of investing in their treatment (ie, smokers enrolling in a clinical trial in which all pharmacotherapy is provided at no cost).

When other significant factors (number of cigarettes smoked per day, initial FTCD result, age at smoking initiation, number of prior attempts to quit) were included in multivariate analysis, a history of depression was not significantly associated with smoking abstinence after 1 year in either men or women. These results support the efficacy of intensive tobacco-dependence treatment in patients who have a history of depression and the use of a program that addresses the needs of each individual patient.

The high prevalence of smoking among patients having practically all types of psychiatric diseases is an indisputable and well-documented fact. On average, smokers who have psychiatric disorders smoke more cigarettes and are more dependent on nicotine (both of which were confirmed by our study) and are more often addicted to other drugs (5,27). In the United States, 44% to 46% of all cigarettes sold are smoked by people who have a psychiatric disease (5,28,29). Clinical practice and epidemiological studies show that the association between smoking and depression has been rising (eg, the Stirling County Study [30]). The relationship between depressive disorders and smoking between 1952 and 1970 was not significant, but in 1992 the probable incidence of a depressive disorder among smokers was 3 times the incidence among nonsmokers (30).

This study has several limitations. The main limitation is that it used a nonrandomized, retrospective design based on chart review and clinical data. As such, we had limited patient contact, and we were unable to assess treatment support outside of the program. In addition, some smokers were unwilling to discuss their prior psychiatric problems during their first visit or tended to trivialize them. This may have led to classification (history of depression vs no history of depression) errors.

Our study results substantiate the need for implementing interventions designed for smoking cessation and providing intensive treatment of tobacco dependence in patients with psychiatric disorders. Much of the current literature concurs that short interventions should be provided for all patients as part of routine treatment and that intensive treatment should be provided whenever a patient shows interest in it.

Data have suggested that rates of smoking abstinence are lower for people who have a history of depression. Our findings suggest that individualized therapy obtained through a clinical tobacco treatment program can potentially mitigate the discrepant abstinence rates between smokers with and without a history of depression. Our next step will be to evaluate rates of depression among patients during implementation of the treatment program.
